# Roles of histamine on the expression of aldehyde dehydrogenase 1 in endometrioid adenocarcinoma cell line

**DOI:** 10.1002/cam4.296

**Published:** 2014-07-10

**Authors:** Yi Wang, Yang Jiang, Jun-ichiro Ikeda, Tian Tian, Atsushi Sato, Hiroshi Ohtsu, Eiichi Morii

**Affiliations:** 1 Department of Pathology, Osaka University Graduate School of MedicineYamada-oka 2-2, Suita, 565-0871, Japan; 2 Department of Pathology, Harbin Medical University of China157 Baojian Road, Harbin, Heilongjiang, 150081, China; 3 Department of Applied Quantum Medical Engineering, School of Engineering, Tohoku University6-6-02-3 Aramaki-aza, Aoba, Sendai, 980-8579, Japan

**Keywords:** Aldehyde dehydrogenase 1, cancer-initiating cells, endometrioid adenocarcinoma, histamine, histamine receptor

## Abstract

Cancer-initiating cells (CICs) are a limited number of cells that are essential for maintenance, recurrence, and metastasis of tumors. Aldehyde dehydrogenase 1 (ALDH1) has been recognized as a marker of CICs. We previously reported that ALDH1-high cases of uterine endometrioid adenocarcinoma showed poor prognosis, and that ALDH1 high population was more tumorigenic, invasive, and resistant to apoptosis than ALDH1 low population. Histamine plays a critical role in cancer cell proliferation, migration, and invasion. Here, we examined the effect of histamine on ALDH1 expression in endometrioid adenocarcinoma cell line. The addition of histamine increased ALDH1 high population, which was consistent with the result that histamine enhanced the invasive ability and the resistance to anticancer drug. Among 4 types of histamine receptors, histamine H1 and H2 receptor (H1R and H2R) were expressed in endometrioid adenocarcinoma cell line. The addition of H1R agonist but not H2R agonist increased ALDH1. The antagonist H1R but not H2R inhibited the effect of histamine on ALDH1 expression. These results indicated that histamine increased the expression of ALDH1 via H1R but not H2R. These findings may provide the evidence for exploring a new strategy to suppress CICs by inhibiting ALDH1 expression with histamine.

## Introduction

Histamine, a biogenic amine, was first identified as an autacoid having potent vasoactive properties 1. Subsequently, it was recognized for its multiple regulatory activities in the immune systems 2–4. Recently, histamine has been demonstrated to play a critical role in cell proliferation and differentiation 5–9, hematopoiesis 4,7, embryonic development 10, regeneration 11, and tumor growth 5,6,12. Of particular note is that histamine has been reported to induce the proliferation and differentiation of hematopoietic and neural stem cells 7–9. Moreover, high histamine biosynthesis and content have been indicated in human tumors including melanoma, lymphoma, leukemia, colon, gynecologic, and breast cancer, as well as in experimental tumors 12–17. Histamine is considered to play a critical role in cancer cell proliferation, migration, and invasion, the tumor microenvironment and immune system responses 5,6,12–17. These various effects of histamine are mediated through the activation of specific histamine receptors (H1R, H2R, H3R, and H4R), which are all G protein-coupled receptors 18,19. Therefore, histamine receptors have been identified as important targets for the treatment of cancers 5,12.

Cancers consist of heterogeneous cell populations derived from a single clone. Recently, it has been demonstrated that cells with tumorigenic potential are limited to a small stem-cell population, called cancer-initiating cells (CICs) 20,21. Thus, eradication of CICs is essential to cure cancers. Several markers have been reported in CICs 22–28. Among these, aldehyde dehydrogenase 1 (ALDH1) is demonstrated to be a general CIC marker of various malignancies, such as leukemia, breast, brain, colon, and lung cancers 28–34. ALDH1 is a cytosolic enzyme responsible for oxidizing a range of aldehydes to their corresponding carboxylic acids, and involves in the degradation of toxins and the self-protection of cells 35. Interestingly, ALDH also involves in the degradation of histamine 36.

We found previously that ALDH1 could be a marker of CICs in uterine endometrioid adenocarcinoma. The population of ALDH1 high cells was more invasive, antiapoptotic, and tumorigenic than the population of ALDH1 low cells in endometrial adenocarcinoma. High activity of ALDH1 was shown to be associated with poor prognosis in these cells 37. In this study, the effects of histamine and histamine receptors on ALDH1 expression were examined in endometrioid adenocarcinoma cell line. We found that histamine enhanced tumor cell invasion ability and resistance against anticancer drug. Meanwhile, histamine via H1R increased ALDH1 expression; while, the antagonist of histamine via H1R inhibited the ALDH1 expression in endometrial adenocarcinoma cell line. This finding suggested that histamine via H1R plays a stimulatory role on CICs in endometrial adenocarcinoma and H1R may be a potential therapeutic target to cure the cancer via eradicating CICs.

## Materials and Methods

### Reagents and cells

Histamine dihydrochloride was purchased from Sigma-Aldrich (St.louis, MO). The agonist of H1R, histamine trifluoromethyl toluidide dimaleate (HTM), the agonist of H2R, dimaprit dihydrochloride (DIM) and the antagonist of H2R, cimetidine (CIM) were purchased from Tocris Bioscience (Minneapolis, MN). The antagonist of H1R, pyrilamine maleate salt (PYR) was purchased from Sigma-Aldrich. HEC-1 endometrioid adenocarcinoma cell line and MCF-7 breast cancer cell line were obtained from the Health Science Research Resources Bank of Osaka, Japan. HMC-1 mastocytoma cell line was kindly provided from Dr. Kitamura (Osaka University, Osaka, Japan). The doses used in these experiments of these agonists and antagonists were adjusted to those in previous reports 8,18,19.

### Matrigel invasion assay

Invasion of tumor cells into Matrigel was examined with a BD BioCoat Matrigel Invasion Chamber (BD Biosciences, Franklin Lakes, NJ). Briefly, HEC-1 cells were seeded in Dulbecco's modified eagle medium (DMEM) without fetal bovine serum (FBS) in the Matrigel invasion upper chamber and cultured for 72 h. The lower chamber contained DMEM supplemented with10% FBS. Invading cells were stained with a Diff quick staining kit (Siemens, Munich, Germany). The number of invading cells was counted in a blinded manner at four microscopic fields per well with a magnification of 20× and the extent of invasion was expressed as the average number of cells per square millimeter.

### Apoptosis-based anticancer drug assay

Cisplatin, an anticancer drug, is commonly used for the treatment of endometrioid adenocarcinoma. Cells (2 × 10^5^) were seeded into 6-well cell culture plates with DMEM-10% FBS and cultured for 18 h at 37°C in 5%CO_2_ condition. Various concentrations of cisplatin (0, 4, 8 *μ*g/mL, Sigma, St Louis, MO) were added, with or without the pretreatment of histamine for 2 h. After 24 h, the apoptosis of cells was assessed with APOPCYTO Annexin V-Azami-Green Apoptosis Detection Kit (MBL, Nagoya, Japan) according to the manufacturer's instruction using FACS Canto II (BD Biosciences).

### Scratch assay

Confluent HEC-1 cells were scraped using sterilized tips, and incubated in culture medium for 24 h. Images were captured at 0 and 24 h after the scratch.

### Aldefluor assay

Cells were cultured in DMEM (Wako, Osaka, Japan) supplemented with 10% FBS (Nippon Bio-Supply Center, Tokyo, Japan). The Aldefluor kit (StemCell Technologies, Vancouver, BC, Canada) was used to evaluate ALDH1 high population, according to the manufacturer's instruction. Briefly, cells were suspended in Aldefluor assay buffer containing the ALDH substrate BODIPY-aminoacetaldehyde and incubated for 45 min at 37°C. The brightly fluorescent ALDH1-expressing cells were detected with FACS Canto II (BD Biosciences). As a negative control, cells were treated with 50 *μ*mol/L diethylaminobenzaldehyde (DEAB). ALDH1 high population was evaluated with or without histamine dihydrochloride and the agonist and antagonist of H1R and H2R. In some experiments, ALDH1 high and ALDH low populations were sorted with FACS Aria II (BD Biosciences).

### Reverse transcription-polymerase chain reaction

Subconfluent cells were harvested, and total RNA was extracted using the RNeasy RNA extraction kit (Qiagen, Germantown, MD). First-strand cDNA synthesis was performed with oligo-(dT) priming with the SuperScript III First-Strand Synthesis System for reverse transcription-polymerase chain reaction (RT-PCR, Life Technologies, Carlsbad, CA). PCR was performed with the cDNA generated using PrimeSTAR™ Max DNA Polymerase (TaKaRa, Shiga, Japan). The primers used were: +5′-CATTCTGGGGGCCTGGTTTCTCT-3′ and −5′-CTTGGGGGTTTGGGATGGTGACT-3′ for human H1R; +5′-CCGGCYCCGCAACCTGAC-3′ and −5′-CTGATCCCGGGCGACCTTGA-3′ for human H2R; +5′-CCACTGTATGTACCCTACGTGCTG-3′ and −5′-ATGCTGAGGTTAAAGAAGGTGACG-3′ for human H3R; +5′-GAATTGTCTGGCTGGATTAATTTGCTAATTTG -3′ and −5′-AAGAATGATGTGATGGCAAGGATGTACC-3′ for human H4R; +5′-AATCTTCAAGCACATGTC-3′ and −5′-CTGGATAGTGGCCGGGATGA-3′ for human l-histidine decarboxylase (HDC). The mixture was subjected to 35 cycles of denaturation at 98°C for 10 sec, annealing at 55°C for 5 sec, and extension at 72°C for 1 min. The PCR products were electrophoresed in 2% agarose gels and visualized by ethidium bromide staining.

### Quantitative RT-PCR

The quantitative RT-PCR (qRT-PCR) was performed with StepOnePlus™ Real-Time PCR instrument (Applied Biosystems, Foster City, CA) using Taqman probe/primer sets specific for human ALDH1A1, H1R, H2R, H3R, and H4R. GAPDH was used as a reference for gene amplification (Applied Biosystems).

### Immunoblotting

Cells were washed with ice-cold phosphate buffered saline, and lysed using the buffer containing 10 mmol/L Hepes, 10 mmol/L KCl, 1 mmol/L ethylene diamine tetaraacetic acid, 0.1 mmol/L dithiothreitol and 10% protease inhibitor cocktail (Sigma). Electrophoresis was performed with 10% sodium dodecyl sulfate–polyacrylamide gels (ATTO, Tokyo, Japan) and proteins were transferred to polyvinylidene fluoride membranes (Millipore, Bedford, MA). Anti-ALDH1 antibody (1000×) and antiactin antibody (Sigma, 1000×) were used as the primary antibody. horse radish peroxidase (HRP)-conjugated anti-mouse IgG (H+L chain) (MBL, 1000×) or HRP-conjugated anti-rabbit IgG (H+L chain) (MBL, 1000×) was used as the secondary antibody.

### Statistical analysis

Statistical analysis for experimental studies was carried out using Student's *t*-tests. The values are shown as the mean ± standard error (SE) of at least three experiments. The *P* ≤ 0.05 was considered as statistical significance.

## Results

### Effect of histamine on HEC-1 endometrial adenocarcinoma cell line in the invasion ability, resistance against anticancer drug, and migration ability

**Figure 1:**
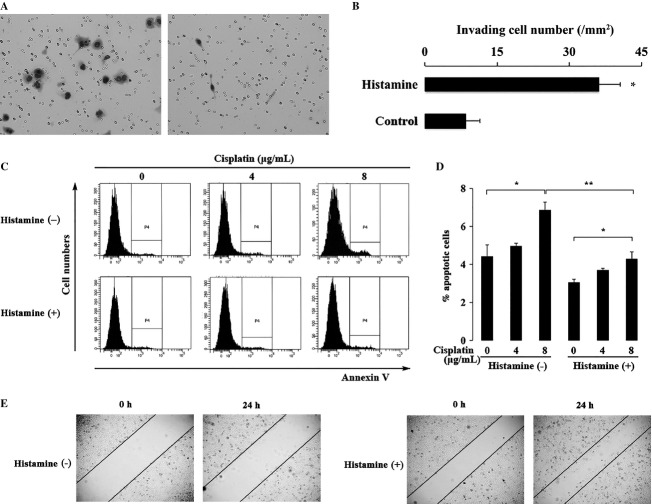
Effects of histamine on cell invasion activity, resistance to the anticancer drugs and migration activity. (A and B) Matrigel invasion assay. HEC-1 cells invaded through Matrigel (20×) (a, with histamine (10 *μ*g/mL); left, and without histamine; right), and the numbers of invading cells per mm^2^ were shown (B). (C) Apoptosis-based anticancer drug assay. Cells were labeled with Annexin δ, and analyzed by flow cytometry. Apoptotic cells of HEC-1 with or without the stimulation of histamine (10 *μ*g/mL) were compared in the presence of various amounts of cisplatin (0, 4, 8 *μ*g/mL). (D) The quantitative results of the apoptotic cells population were shown in bar graph. *P < 0.05 when compared to the value without cisplatin. **P < 0.05 when compared to the value without histamine. (E) Scratch assay was performed in the presence or absence of histamine (10 *μ*g/mL, 0.25×).

Histamine has been reported to be involved in cancer cell migration and invasion 5,12. Here, to examine the effect of histamine on HEC-1 endometrial adenocarcinoma cell line in the invasion ability, matrigel invasion assay was performed. When HEC-1 cells were incubated with histamine for 2 h, the number of invading cells was higher than that of histamine-untreated cells. The result indicated that histamine-treated cells possessed stronger invasive ability than untreated cells (Fig.1A and B).

To examine the effect of histamine on the resistance against anticancer drug, cisplatin was added to cells with or without the pretreatment of histamine for 2 h. Cells without the pretreatment of histamine were more vulnerable to cisplatin than those pretreated with histamine (Fig.1C and D). These results indicated that histamine enhanced the resistance to anticancer drug in HEC-1.

Moreover, the effect of histamine on migration ability was examined by scratch assay. In the presence of histamine, the number of migrating cells at 24 h after scratch was higher than that without histamine (Fig.1E).

### Effect of histamine on the expression of ALDH1

**Figure 2:**
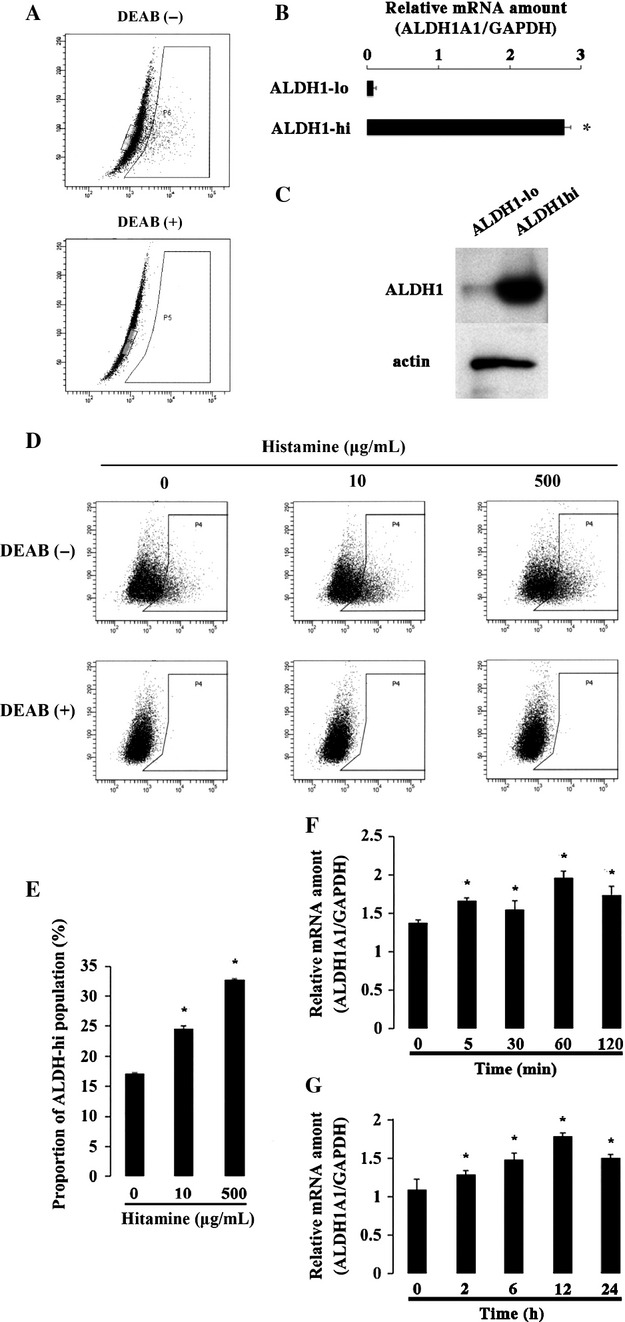
Effect of histamine on the proportion of ALDH high cells and ALDH1 mRNA expression. (A) Aldefluor assay was performed. ALDH1 hi cells were shown in the right box, and ALDH1 lo cells in the left box. (B) Quantification of ALDH1A1 gene expression in the sorted ALDH1 lo and ALDH1 high cells. *P < 0.05 when compared to the value of ALDH1-low. (C) Immunoblotting of ALDH1 and actin proteins in the sorted ALDH1 low and ALDH1 high cells. (D) The proportion of ALDH high cells was determined by Aldefluor assay. HEC-1 cells were treated with histamine (10 *μ*g/mL and 500 *μ*g/mL) for 2 h. (E) The quantitative results of Aldefluor assay were shown in bar graph. *P < 0.05 when compared to the value without histamine. (F) Quantification of ALDH1A1 gene expression. HEC-1 cells were treated with histamine (10 *μ*g/mL) in the indicated duration (5, 30, 60, and 120 min), and mRNA was extracted. *P < 0.05 when compared to the value without histamine. (G) In separate experiments, HEC-1 cells were treated with histamine (10 *μ*g/mL) in the longer duration time (2, 6, 12, and 24 h), and mRNA was extracted. *P < 0.05 when compared to the value without histamine.

ALDH1 activity was examined with Aldefluor assay. The brightly fluorescent cells were detected in the absence of DEAB but disappeared in the presence of DEAB (Fig.2A). When sorted, the brightly fluorescent cells expressed higher amount of ALDH1 mRNA and higher amount of ALDH protein as compared to the nonfluorescent cells (Fig.2B and C). Then, the fluorescent cells were ALDH-high and the nonfluorescent cells ALDH-low. To examine the effect of histamine on ALDH1 activity, HEC-1 cells were treated with histamine for 2 h at two different concentrations. With the treatment of histamine, the proportion of ALDH1 high population increased in a dose-dependent manner (Fig.2D and E). The qRT-PCR was performed to detect whether histamine regulated ALDH1 expression in mRNA level. When histamine was added, the mRNA level of ALDH1 increased significantly already at 5 min, and its increase was relatively steady during the observed period (5 min to 24 h) (Fig.2F and G).

### Expression of histamine receptors

**Figure 3:**
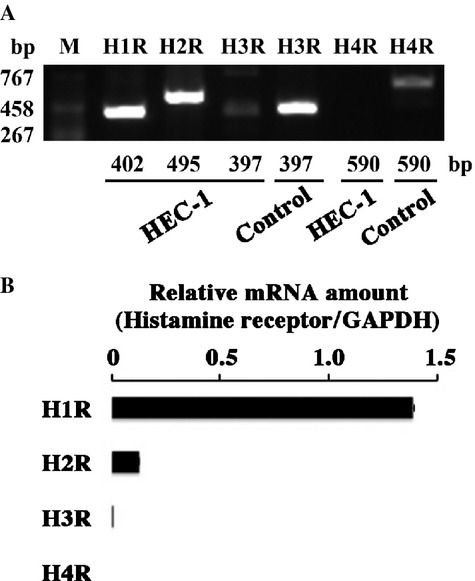
Expression of histamine receptors in HEC-1 endometrioid adenocarcinoma cell line. (A) H1R, H2R, H3R, and H4R mRNA expression in HEC-1 endometrioid adenocarcinoma cell line were detected by RT-PCR. As a positive control for H3R and H4R mRNA expression, RNA obtained from MCF-7 and HMC-1 was used, respectively. (B) Quantification of four subtypes of histamine receptors' gene expression.

As four kinds of histamine receptors have been reported 18–20, the effect of histamine on HEC-1 cells were thought to be mediated through those receptors. RT-PCR revealed that H1R and H2R were expressed in HEC-1 (Fig.3A). In contrast, the expression of H3R and H4R was hardly detected. These results were confirmed by qRT-PCR (Fig.3A and B).

### Effect of agonists and antagonists of H1R and H2R on ALDH1

**Figure 4:**
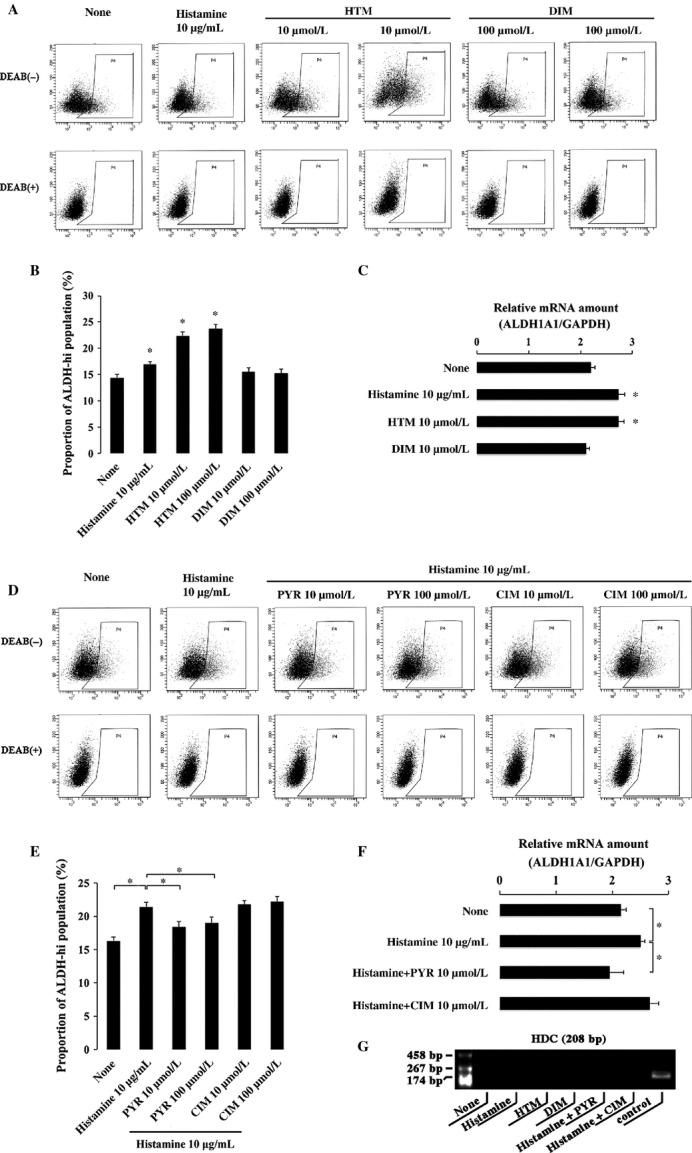
Effect of the agonists and antagonists of H1R and H2R on the expression of ALDH1 in HEC-1 endometrioid adenocarcinoma cell line. (A) The proportion of ALDH high cells was determined by Aldefluor assay. HEC-1 cells were treated with histamine, the agonist of H1R (HTM) and the agonist of H2R (DIM) for 2 h, respectively. (B) The quantitative results of Aldefluor assay were shown in bar graph. *P < 0.05 when compared to the value with neither histamine nor the agonists of H1R and H2R. (C) Quantification of ALDH1A1 gene expression. HEC-1 cells were treated with histamine, the agonist of H1R (HTM) and the agonist of H2R (DIM) for 2 h, respectively. mRNA was extracted for RT-PCR analysis. *P < 0.05 when compared to the value with neither histamine nor the agonists of H1R and H2R. (D) The proportion of ALDH high cells was determined by Aldefluor assay. HEC-1 cells were treated with histamine, histamine with the antagonist of H1R (PYR) and histamine with the antagonist of H2R (CIM) for 2 h, respectively. (E) The quantitative results of Aldefluor assay were shown in bar graph. *P < 0.05 (F) Quantification of ALDH1A1 gene expression. HEC-1 cells were treated with histamine, histamine with the antagonist of H1R (PYR), and histamine with the antagonist of H2R (CIM) for 2 h, respectively. *P < 0.05. (g) Expression of HDC mRNA was examined with RT-PCR in HEC-1 cells in the absence or presence of histamine (10 *μ*g/mL), HTM, DIM, histamine + PYR, and histamine + CIM. As a positive control for HDC mRNA expression, RNA obtained from HMC-1 was used.

To examine whether the effect of histamine on ALDH1 expression was mediated by H1R and H2R, the agonists and antagonists were used. HTM and DIM are agonists of H1R and H2R, respectively. PYR and CIM are antagonists of H1R and H2R, respectively. The addition of HTM but not DIM increased the proportion of ALDH1 high population and the ALDH1 mRNA level (Fig.4A, B, and C). The addition of PYR but not CIM inhibited the effect of histamine on ALDH1 activity (Fig.4D, E, and F). These results indicated that H1R but not H2R mediated the effect of histamine on ALDH1. We confirmed that no expression of HDC was detected in HEC-1 cells under any conditions (Fig.4G).

### Effect of H1R agonist HTM on HEC-1 endometrial adenocarcinoma cell line in the invasion ability, resistance against anticancer drug, and migration ability

**Figure 5:**
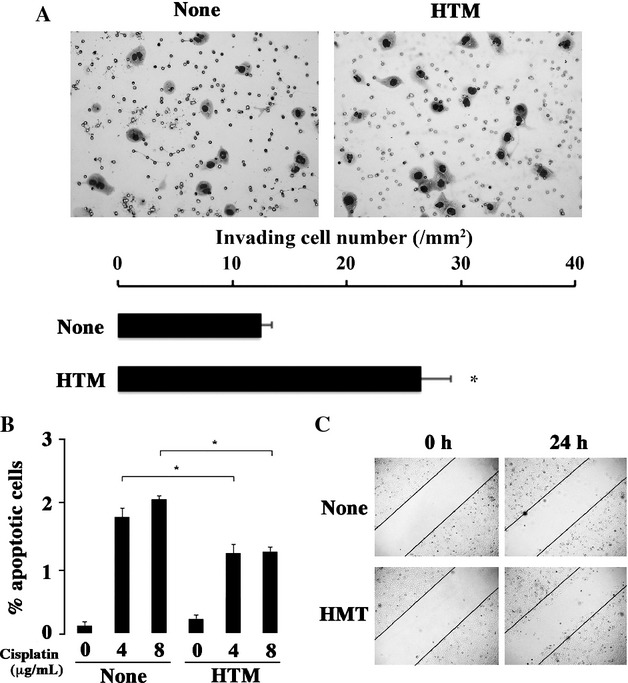
Effect of HTM on the invasion activity, resistance to the anticancer drugs and migration activity. (A) Matrigel invasion assay. HEC-1 cells invaded through Matrigel (20×) without (left) and with HTM (right), and the numbers of invading cells per mm^2^ were shown. (B) Apoptosis-based anticancer drug assay. Cells were labeled with Annexin *δ*, and analyzed by flow cytometry. Apoptotic cells of HEC-1 without and with HTM were compared in the presence of various amounts of cisplatin (0, 4, 8 *μ*g/mL). The quantitative results of the apoptotic cells population were shown in bar graph. *P < 0.05 when compared to the value without HTM. (C) Scratch assay was performed without and with HTM (0.25×).

When HTM was added, the number of invading cells was higher than that of untreated cells (Fig.5A). The addition of HTM reduced the apoptotic cells induced by cisplatin (Fig.5B), indicating that the resistance to cisplatin was mediated by the H1 receptor. Moreover, the migrating cell number increased when HTM was added (Fig.5C).

## Discussion

Targeting CICs are important for the treatment of cancers, because CICs are considered to be responsible for the maintenance, recurrence, and metastasis 38,39. Considerable evidence indicates that ALDH1 activity serves as a valuable functional marker for the identification of CICs. ALDH1-high expressing tumor cells are tumorigenic and resistant to chemotherapy 40–42. In the clinical studies, increased ALDH1 activity is correlated with metastasis and poor prognosis in several types of tumors 28–34. Based on these reports, we previously demonstrated that ALDH1 expression was correlated to size of tumor, lymphatic invasion, recurrence, and prognosis of patients in uterine endometrioid adenocarcinoma. The population of ALDH1 high cells was more invasive, antiapoptotic, and tumorigenic than the population of ALDH1 low cells in HEC-1 endometrioid adenocarcinoma cell line, in which abundant ALDH high cells were detected by Aldefluor assay as compared to other endometrioid adenocarcinoma cell lines 37. Here, we focused on potential factors regulating ALDH1 expression. Histamine enhances migration in several normal cells, such as eosinophil, dendritic cells, and lung fibroblast 43–45. In addition, histamine induces the proliferation and differentiation of hematopoietic and neural stem cells 7–9. Since the first report in 1984 showing that the inhibition of the histamine-synthesizing enzyme, HDC which catalyzes the biosynthesis of histamine from histidine, resulted in antitumoral effects on experimental tumors in rodents 46, a large body of experimental evidence has supported the critical role of HDC and histamine in tumor development and progression 5,6,12–17. In diverse human tumors, histamine concentration is higher than that of surrounding normal tissue 15,17,47. Moreover, histamine has been reported to be involved in cancer cell proliferation, migration and invasion, tumor microenvironment, and immune system responses in diverse human tumors including melanoma, lymphomas, leukemia, colon, gynecologic and breast cancer, as well as in experimental tumors 12–17. Similar with these reports, our findings suggested that histamine appeared to play a tumorigenic role in endometrioid adenocarcinoma cell line. These batteries of evidence prompted us to investigate the correlation of histamine and ALDH1 expression. We showed that histamine increased the proportion of ALDH1 high cells in endometrioid adenocarcinoma cell line. Since ALDH1 expression is supposed to be correlated to CIC function, 28–37 histamine might possess stimulatory effect on CICs.

It is known that histamine exerts its effect through the activation of four kinds of specific histamine receptors designated H1R–H4R. These four receptors exhibit various effects 48. Recent studies have shown that the four subtypes of histamine receptors have varying degrees of expression in different forms of cancers 5. Here, we found that H1R and H2R were highly expressed in HEC-1 endometrioid adenocarcinoma cell line. Moreover, only H1R mediated the effect of histamine on the proportion of ALDH1 high population and ALDH1 mRNA expression. Therefore, H1R was suggested to be an effective and prompt “switch” to the regulation of ALDH1 expression and the proportion of CICs in endometrioid adenocarcinoma cell line. Various antagonists of H1R might be effective adjuvant therapeutics to eradicate CICs.

Taken together, the present study is the first to demonstrate the effect of histamine and H1R on ALDH1 expression in endometrioid adenocarcinoma. As ALDH1 activity is supposed to be a general CIC marker of various tumors 28–34,37, this finding provides an adjuvant therapeutic approach to eradicate CICs and cure the cancer. Further studies are necessary to clarify the effect of histamine receptors on the other types of CICs and the precise mechanism of histamine activity.
